# Machine Learning Tool for New Selective Serotonin and Serotonin–Norepinephrine Reuptake Inhibitors

**DOI:** 10.3390/molecules30030637

**Published:** 2025-01-31

**Authors:** Natalia Łapińska, Jakub Szlęk, Adam Pacławski, Aleksander Mendyk

**Affiliations:** 1 Department of Pharmaceutical Technology and Biopharmaceutics, Jagiellonian University Medical College, 30-688 Kraków, Poland; natalia.czub@uj.edu.pl (N.Ł.); adam.paclawski@uj.edu.pl (A.P.); aleksander.mendyk@uj.edu.pl (A.M.); 2 Bioinformatics and In Silico Analysis Laboratory, Center for the Development of Therapies for Civilization and Age-Related Diseases (CDT-CARD), 8 Skawińska St., 31-066 Kraków, Poland

**Keywords:** depression, QSAR model, SERT, NET, artificial Intelligence, SerotoninAI

## Abstract

Depression, a serious mood disorder, affects about 5% of the population. Currently, there are two groups of antidepressants that are the first-line treatment for depressive disorder: selective serotonin reuptake inhibitors and serotonin–norepinephrine reuptake inhibitors. The aim of the study was to develop Quantitative Structure–Activity Relationship (QSAR) models for serotonin (SERT) and norepinephrine (NET) transporters to predict the affinity and inhibition potential of new molecules. Models were developed using the Automated Machine Learning tool Mljar based on 80% of the dataset according to 10-fold cross-validation and externally validated on the remaining 20% of data. The molecular representation featured two-dimensional Mordred descriptors. For each model, Shapley additive explanations analysis was performed to clarify the influence of the descriptors on the models’ predictions. Based on the final QSAR models, the following results were obtained: NET and pIC50 value RMSE_test_ = 0.678, R^2^_test_ = 0.640; NET and pKi RMSE_test_ = 0.590, R^2^_test_ = 0.709; SERT and pIC50 RMSE_test_ = 0.645, R^2^_test_ = 0.678; SERT and pKi value RMSE_test_ = 0.540, R^2^_test_ = 0.828. QSAR models for serotonin and norepinephrine transporters have been made available in a new module of the SerotoninAI application to enhance usability for scientists.

## 1. Introduction

Mental health is a key aspect of well-being that can affect daily life. Among various mental disorders, one of the most common is depression, which is also known as depressive disorder. According to the World Health Organization, around 280 million people suffer from it with women affected more frequently than men [1]. Depression is characterized as low mood, loss of pleasure and interest in activities for long period of time, which affects personal and professional life [2]. Untreated depression can tragically lead to suicide, emphasizing the critical need for effective treatment of the disease. In recent years, treatment of depression is based on drugs with different mechanisms of action. However, a group of drugs used as first-line treatment is selective serotonin reuptake inhibitors (SSRIs), e.g., Fluoxetine, Paroxetine, and Sertraline. By inhibiting the serotonin transporter, they increase the concentration of serotonin in the synaptic cleft. In case of a low treatment efficacy of SSRIs, Duloxetine, Venlafaxine or Milnacipran, from the serotonin and norepinephrine reuptake inhibitor (SNRI) group is prescribed for a patient. SNRI increases the concentration of these two neurotransmitters, acting to improve the patient’s mood [3,4]. Due to the importance of serotonin (SERT) and norepinephrine (NET) transporters in the mechanism of action of the most commonly used antidepressants, they form the foundation of the conducted studies presented in this manuscript.

The significant development of computational methods, especially machine learning, allows the search for advanced relationships between the structure of ligands and activity toward the selected protein. Such dependencies, called QSAR (Quantitative Structure–Activity Relationship) models, enable the discovery of new molecules. QSARs are used to find new molecules that interact with a chosen biological target with high activity [5,6].

The serotonin transporter in terms of QSAR model analysis or development has been a foundation of research over the past 20 years. In the first paper that appeared in 2004, Kulkarni et al. described N-substituted 3-alpha-(bis[4-fluorophenyl]methoxy)tropane derivatives for 76 molecules [7]. In subsequent years, studies including N- arylmethylpiperidinamine [8], phenylpiperidine [9] and phenethylamine [10] derivatives were published. The size of the databases ranged from 7 to 213 serotonin transporter ligands [7,8,9,10,11,12,13,14,15,16,17,18,19,20,21,22]. On the other hand, QSAR models for the norepinephrine transporter were rarely the centerpiece of published studies. Usually, these papers were part of the serotonin transporter-related work already presented above [7,8,10,13,16,20]. The article that focused exclusively on the norepinephrine transporter is a paper by Olasupo et al. presenting a study for a set of 50 ligands using statistical analysis by genetic function approximation (GFA) to develop a QSAR model [23]. In addition, the authors of the above studies were concerned with various forms of measuring the activity of ligands against the selected transporter; these were an inhibition constant Ki [8,13,17,22] and its negative logarithm—pKi (affinity value) [14,16,20], a half maximal effective concentration (EC50) [15] or a half maximal inhibitory concentration (IC50) [21] and the negative logarithm of its concentration—pIC50 [9,18].

A review of the literature reveals a lack of general, cutting-edge models using advanced machine learning techniques that can predict the activity of previously unseen molecules with low error. In a previous paper presenting the application of SerotoninAI, we presented a model predicting the affinity of ligands for the serotonin transporter (pKi value predictions) [24]. In the present study, we wanted to extend this database for serotonin transporter and pKi values with additional data sources. Focusing on the mechanism of action of selective serotonin inhibitors and serotonin–norepinephrine inhibitors, we also created an affinity model for the norepinephrine transporter. The next two QSAR models focused on the level of inhibition of the two transporters. The final result of the study was the development of four QSAR models for the serotonin and norepinephrine transporter and their relevant properties.

In the subsequent sections of the manuscript, we present the obtained results, data acquisition and selection process, a description of the methodology for developing QSAR models along with an analysis of features’ effect on prediction using SHAP analysis.

## 2. Results

### 2.1. Databases

**Figure 1:**
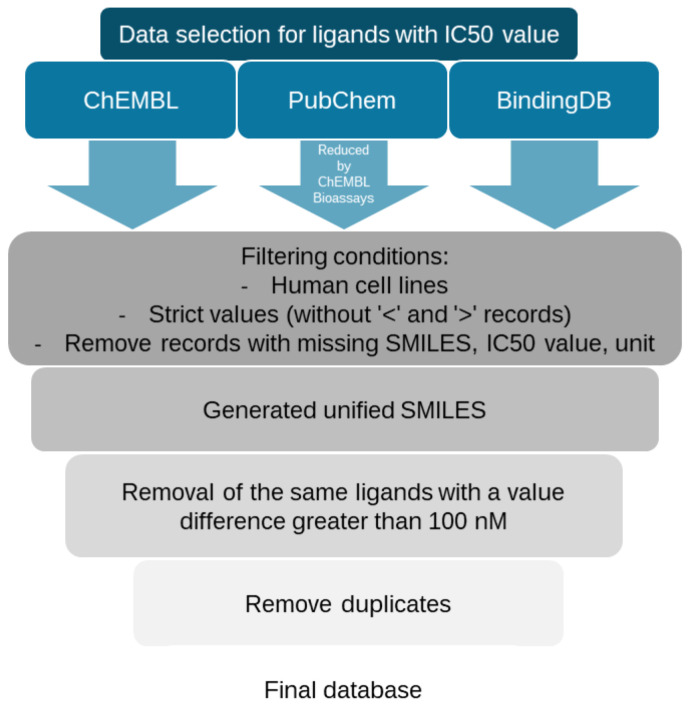
Overview of data selection process for IC50 value for serotonin and norepinephrine transporters.

**Figure 2:**
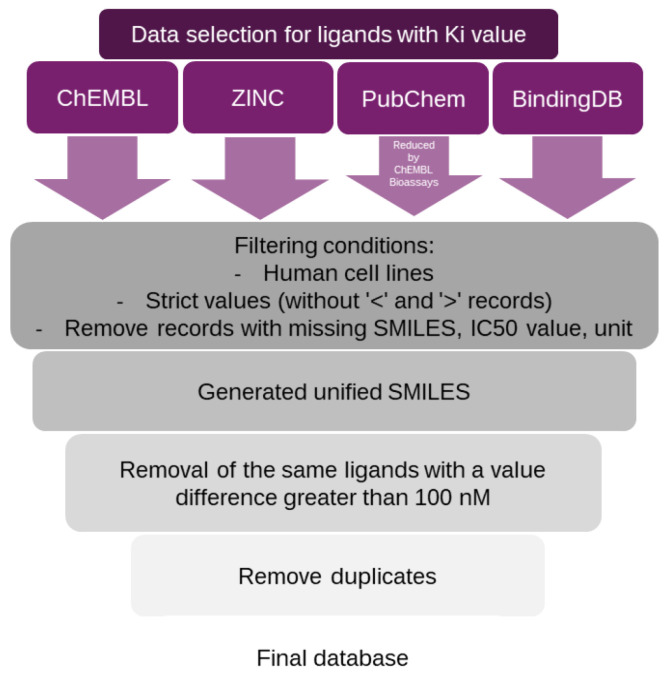
Overview of data selection process for Ki value for serotonin and norepinephrine transporters.

The first step was to design four databases. Figure 1 and Figure 2 below show the overview method of data curation, and Table 1 shows the ligand abundance. The data sources used in this research contained molecules that were found also in other databases.

The study used four open databases as a source of molecule data: BindingDB [25], ChEMBL [26], PubChem [27] and ZINC [28]. However, the ZINC database contains molecule affinity values not inhibitory concentrations. Moreover, all data sources contain overlapping molecules. The BindingDB database contained a high percentage of molecules with the database identifier ChEMBL and ZINC. The PubChem database, in nearly all cases, references ChEMBL as its primary data source. Since the ChEMBL database was accessed separately for this study, duplicating data retrieval from PubChem was deemed unnecessary (accessed on 31 October 2024).

The next database cleaning step included the removal of records with “>” and “<” tags, missing activity values or units, missing SMILES (Simplified Molecular Input Line Entry System), data from studies using non-human cell lines, a set of the same molecules between which differences in Ki or IC50 values exceeded 100 nM, and duplicate molecules based on a unified SMILES generation system. For the serotonin transporter and pKi values, we used our previously curated database [24], which contained 8091 unique molecules, as the primary database. As an additional value, we provided new molecules from the BindingDB and PubChem databases. This yielded an additional 781 molecules. Final databases are available in Supplementary Materials S1–S4.

**Figure 3:**
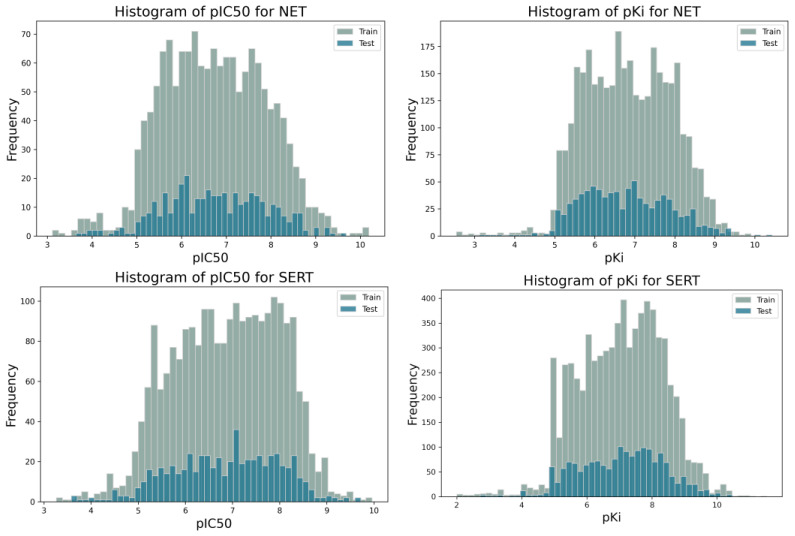
Histograms for training and test sets for biological targets and their corresponding properties.

**Figure 4:**
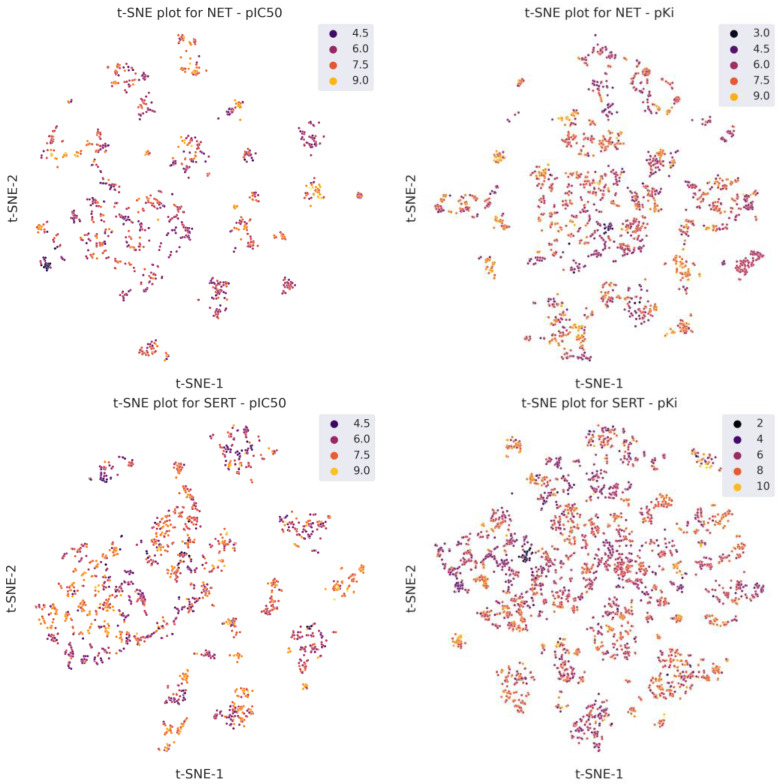
Scatter plots of t-SNE analysis for four databases.

Divided into training and test sets in a ratio of 80:20, the datasets had a similar distribution of the dependent variable, as seen in the histograms shown in Figure 3. The obtained databases are characterized by diverse molecules. Figure 4 shows the results of a t-SNE analysis performed using ChemPlot [29], which is a Python package designed to visualize molecular diversity based on SMILES structures. The t-SNE algorithm is a dimensionality reduction technique that projects multidimensional data into a two-dimensional space. The results presented here show the high diversity of molecules in the databases.

### 2.2. QSAR Models

The best-performing models and their metric values are summarized in Table 2. The recap includes the root mean square error (RMSE) and coefficient of determination R^2^ values for both the training set according to 10-fold cross validation and the test set.

#### 2.2.1. Norepinephrine Transporter—pIC50

**Figure 5:**
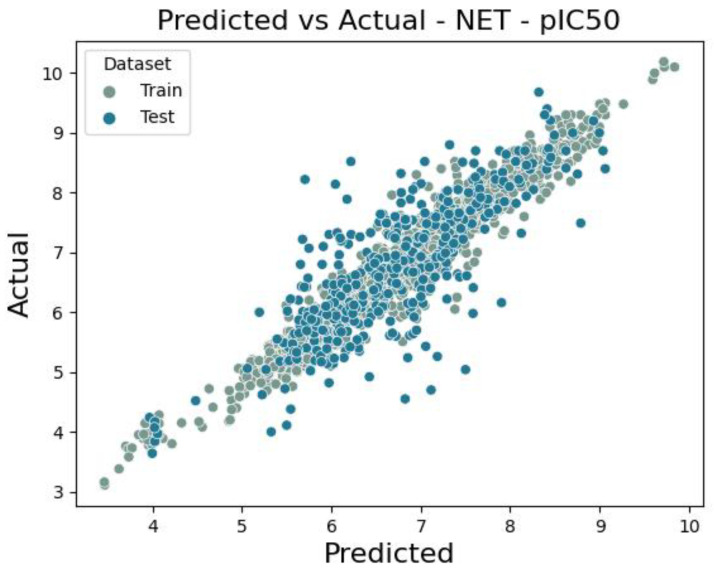
Scatter plot of a comparison of actual vs. predicted values for noradrenaline transporter of pIC50 value.

**Figure 6:**
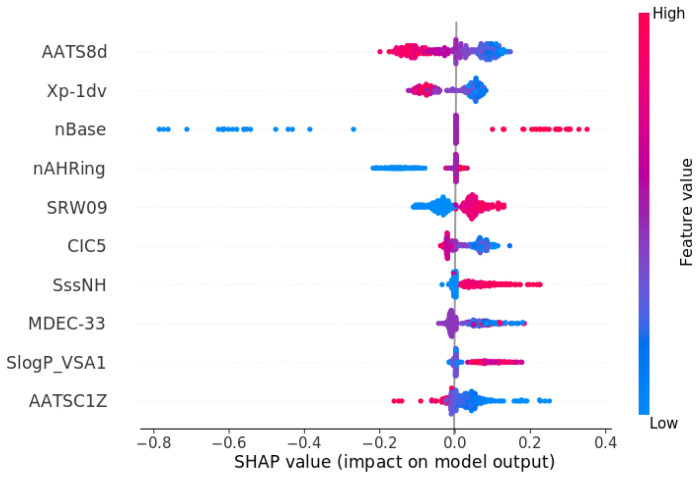
Summary plot of SHAP analysis for ten of the most important descriptors for NET pIC50 QSAR model.

For the database built up with 1879 unique molecules, a QSAR model was developed with RMSE values equal to 0.621 and 0.678 for training and test sets, respectively, where the coefficient of determinations were 0.708 (train set) and 0.640 (test set). Model performance is presented as a scatter plot of predicted versus true values for both the training and test sets (Figure 5). For the obtained ensemble model (2 × Xgboost, 5 × LightGBM, Neural Network, 3 × CatBoost), SHAP analysis was performed based on 20% of the training dataset. Table 3 presents the values for top ten molecular descriptors for the model. Moreover, Figure 6 presents the impact of descriptor values on the derivative of half maximal inhibitory concentration (pIC50).

The most relevant descriptor for predicting pIC50 values for the norepinephrine transporter is the AATS8d descriptor, which, according to Mordred’s documentation [30], is described as the averaged Moreau–Broto autocorrelation of lag 8 weighted by sigma electrons. This two-dimensional feature of ligands at low values has a positive effect on pIC50 values, while at high values, it has a decreasing effect on pIC50. Similar behavior can be observed for the Xp-1dv descriptor (1-ordered Chi path weighted by valence electrons), which describes molecular branching [31]. A high number of base groups (nBase) positively increases the inhibitory activity of ligands, while lower pIC50 values are observed at low ones. This principle can be seen in the case of Milnacipran, where the structure of the drug includes both a primary amine and a tertiary amine (N, N-diethyl), contributing to its interaction at the binding site and its inhibitory activity [32]. For a low number of aromatic rings with a heteroatom (nAHRing), a negative effect on the predicted value is observed, but it cannot be said that a molecule with a number of such rings increases the pIC50 value. The fifth descriptor relevant to this analysis is descriptor SRW09 (walk count (leg-9, only self-returning walk)). It was described in 1993 [33] and for the inhibitory properties of the norepinephrine transporter; high values of it positively affect pIC50 values, and correspondingly low values decrease this dependent variable. The CIC5 descriptor (five-ordered complementary information content) at low values has a positive effect on the pIC50 value. On the other hand, high values have no significant effect, which could be described as neutral. This neutral effect is also observed at low values of the descriptor SssNH (descriptor describing the sum of secondary amines) [34]. On the other hand, high values of the sum of the nitrogen atom have an increasing effect on the value of the derived inhibitory concentration. The molecular distance between tertiary carbon atoms (MDEC-33) is difficult to interpret unambiguously. In contrast, the descriptor SlogP_VSA1 positively influences pIC50 values at high descriptor values. The descriptor SlogP_VSA relates to the molecular property of hydrophobicity and its spatial distribution across a ligand’s surface area. It could link to how the norepinephrine transporter binding site interacts with ligands. The last descriptor, AATSC1Z, mostly positively affects pIC50 values at low values and negatively at high values.

#### 2.2.2. Norepinephrine Transporter—pKi

**Figure 7:**
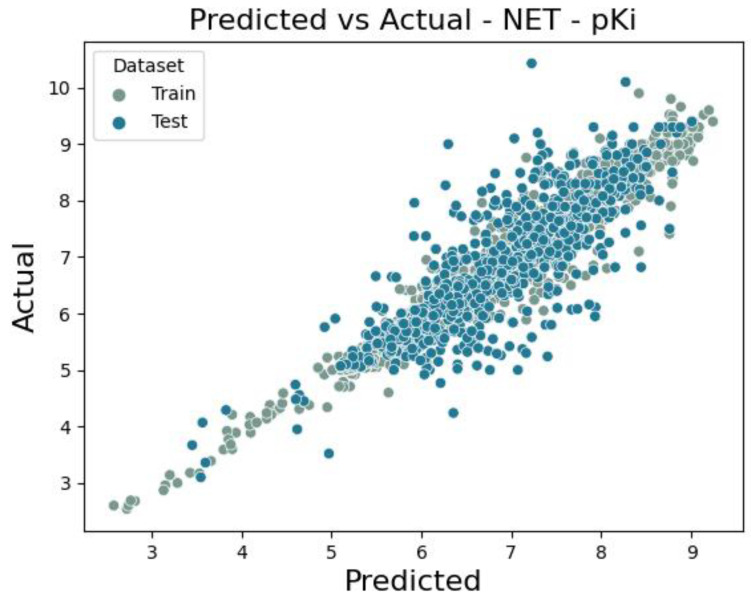
Scatter plot of a comparison of actual vs. predicted values for noradrenaline transporter of pKi value.

**Figure 8:**
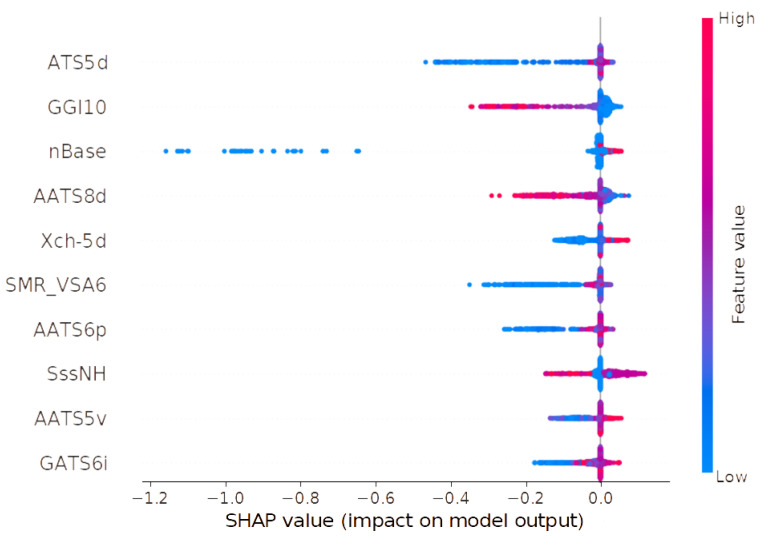
Summary plot of SHAP analysis for ten of the most important descriptors for NET pKi QSAR model.

The number of norepinephrine transporter ligands based on pKi values is 4304. The final ensemble model (3 × Xgboost, 2 × LightGBM, 3 × Neural Network, CatBoost) results of metrics are for the training set RMSE = 0.522 and R^2^ = 0.764. Interestingly, for the test set, the error value is lower, RMSE = 0.590 and R^2^ = 0.709. The performance of the model is visualized as a scatter plot of predicted and actual values for both the training and test sets (Figure 7). Similar to the model predicting pIC50 values, SHAP analysis was also performed. The results are shown in Table 4 and Figure 8.

The SHAP analysis revealed significant relationships between specific descriptors and the affinity values. The ATS5d descriptor, representing the Moreau–Broto autocorrelation of lag 5 weighted by sigma electrons, was identified as the most influential variable in the QSAR model. Lower values of ATS5d were associated with a reduction in pKi, while higher values did not exhibit a clear positive or enhancing effect on affinity. The second descriptor, GGI10, corresponds to the 10-ordered raw topological charge and demonstrated a contrasting pattern. High values of this descriptor were found to negatively influence pKi, whereas low values exhibited a neutral or slightly positive effect. This behavior aligns with findings reported by Olasupo et al. [23], where this descriptor also contributed negatively to the calculated pKi values for NET. For the nBase descriptor, which reflects the count of basic groups in the molecule, low values showed both neutral and negative effects on pKi, while higher values were associated with a minimal increase in affinity. Similarly, the AATS8d descriptor, representing the averaged Moreau–Broto autocorrelation of lag 8 weighted by sigma electrons, was observed to negatively impact pKi at high values, while low values were associated with neutral or marginally positive effects. In the case of Xch-5d, a five-ordered Chi chain weighted by sigma electrons, low values correlated with reduced pKi, whereas higher values positively influenced ligand affinity. The SMR_VSA6 descriptor, a MOE-type variable based on Wildman–Crippen molar refractivity and surface area contributions, exhibited a complex pattern. Low values of SMR_VSA6 had a negative impact on pKi, while higher values were largely neutral with some indication of a slight increase in affinity. The AATS6p descriptor, an averaged Moreau–Broto autocorrelation of lag 6 weighted by polarizability, showed a similar trend to SMR_VSA6. Low values were associated with a reduction in pKi, whereas high values resulted in neutral or slightly positive effects. The SssNH descriptor, an atom-type e-state representing the sum of ssNH, presented an ambiguous relationship with pKi. While high values exhibited both negative and positive influences, low values predominantly showed a neutral effect. It is worth noting that the ssNH atom type refers to nitrogen atoms bonded by two single bonds. For AATS5v, an averaged Moreau–Broto autocorrelation of lag 5 weighted by van der Waals volume, low values were associated with a negative effect on pKi. High values, in contrast, were neutral or contributed to a slight increase in affinity. Finally, the GATS6i descriptor, a Geary coefficient of lag 6 weighted by ionization potential, demonstrated no consistent relationship with pKi. Low values were primarily linked to a negative effect, with occasional neutrality, while high values ranged from slightly negative to marginally positive influences on affinity. Interestingly, for the model predicting pIC50 values for NET, the SHAP analysis similarly identified nBase and AATS8d as significant descriptors, displaying a comparable influence to that observed in the pKi model. However, the SssNH descriptor did not exhibit the same pattern in its effect on pIC50 values, indicating variability in its contribution across different predictive models [30,34].

#### 2.2.3. Serotonin Transporter—pIC50

**Figure 9:**
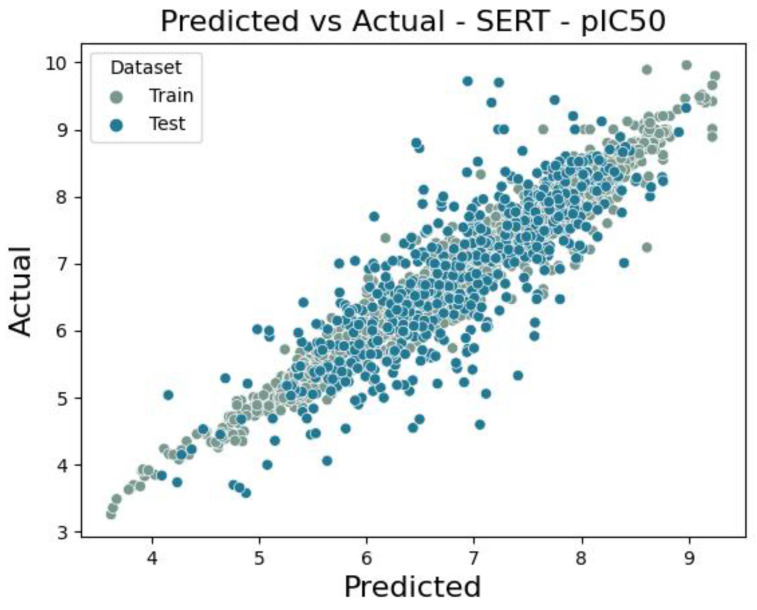
Scatter plot of a comparison of actual vs. predicted values for serotonin transporter of pIC50 value.

The number of serotonin transporter unique ligands with pIC50 values was 2952. The model developed was also an ensemble model built with three types of machine learning algorithms (Xgboost, 4 × LightGBM, 4 × Neural Network). The RMSE values were 0.597 and 0.645 for the training set and test set, respectively. The coefficient of determination, in turn, had values of 0.715—train set and 0.678—test set. The scatter plot in Figure 9 depicts the model’s performance by showing predicted versus true values for the training and test sets.

**Figure 10:**
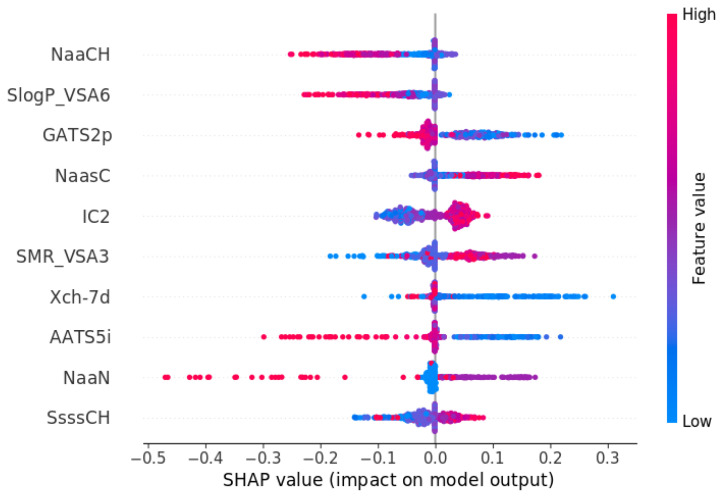
Summary plot of SHAP analysis for ten of the most important descriptors for SERT pIC50 QSAR model.

The second stage of the QSAR model analysis was to analyze the significance of the descriptors affecting the pIC50 value. In Table 5 and Figure 10, we presented a summary of the ten most important descriptors. The most significant descriptor is the number of aromatic carbon atoms of NaaCH. At high values of this descriptor, a decreasing effect on predicted pIC50 values is observed, but at low values, the effect is ambiguous. The same situation is observed for the second descriptor SlogP_VSA6 (logP value and the influence of surface area). The third descriptor GATS2p at low values positively affects the value of pIC50, and high values lower the value of the inhibition derivative. The number of carbon atoms in the aromatic ring additionally forming a single bond (NaasC) at high values positively affects the pIC50 value. On the other hand, a decreasing or neutral effect is seen at low values of NaasC. Neighborhood information content descriptor 2 (IC2) at low values has a negative effect, and at high values, it has a positive effect. SMR_VSA3, described as Wildman–Crippen molar refractivity and surface area, at high values has a positive effect on the pIC50 value but no clear effect at low values. Low values of Chi descriptor (Xch-7d) and AATS5i (average Autocorrelation of Topological Structure descriptor) have an effect on increasing the derived inhibition. The last two descriptors, NaaN (aromatic nitrogen atom) and SsssCH (sum of tertiary carbon atoms), do not represent unambiguous influences. In the case of the aromatic nitrogen atom, a characteristic negative effect of high descriptor values on the reduction in pIC50 values can be observed [30,34].

#### 2.2.4. Serotonin Transporter—pKi

**Figure 11:**
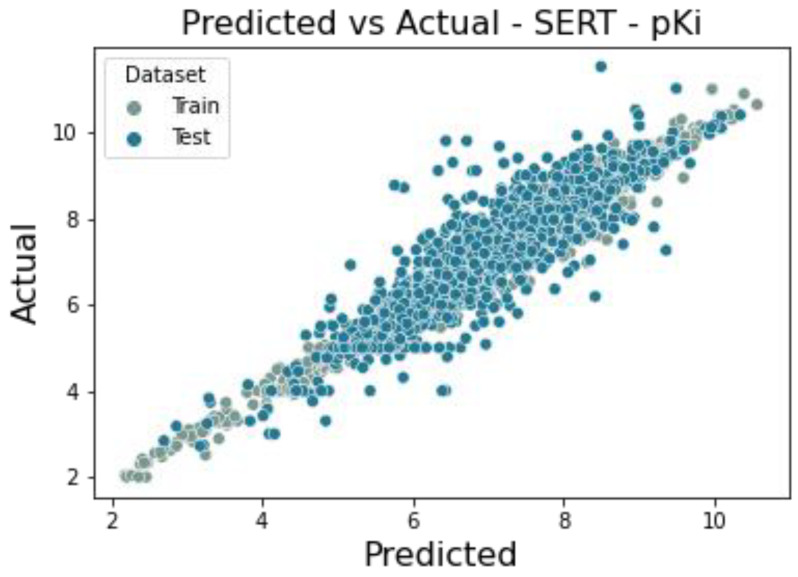
Scatter plot of a comparison of actual vs. predicted values for serotonin transporter of pKi value.

The database containing the serotonin transporter ligands and affinities is represented by 8872 unique molecules. The error values of the ensemble model created with Mljar (3 × Xgboost, 7 × LightGBM, 6 × Neural Network, 2 × CatBoost) are 0.474 and 0.540, and the coefficient of determination is 0.863 and 0.828 for the training and test set, respectively. Model performance is illustrated below using a scatter plot comparing predicted and true values for both the training and test sets (Figure 11).

**Figure 12:**
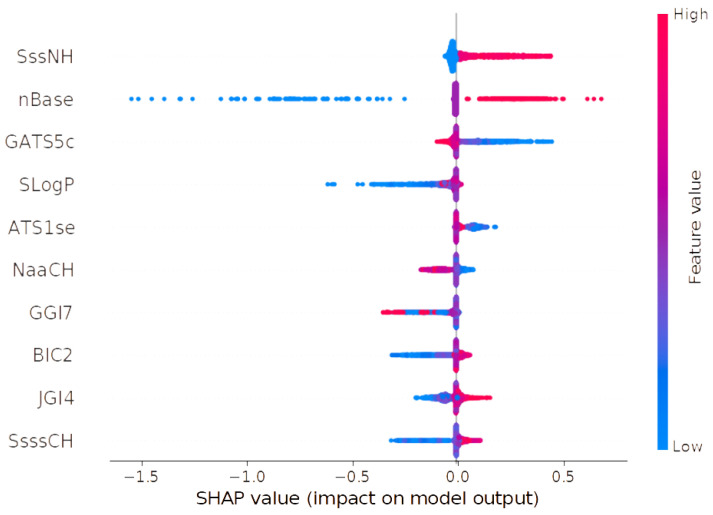
Summary plot of SHAP analysis for ten of the most important descriptors for SERT pKi QSAR model.

SHAP analysis identified a set of most important descriptors; the ten most important are shown in Table 6 and Figure 12 below. The most important descriptor, SssNH, representing the sum of nitrogen atoms bonded by two single bonds, was found to positively influence pKi at high values. Low values, in contrast, showed a slightly negative or neutral effect. Similarly, the nBase descriptor, which quantifies the number of basic groups in a molecule, exhibited a dual influence. Low values corresponded to a negative effect on pKi, while high values were associated with a positive impact. The GATS5c descriptor, a Geary coefficient of lag 5 weighted by Gasteiger charge, showed a nuanced pattern. Low values had a positive or neutral effect, whereas high values were generally neutral with a slight negative influence on pKi. The SLogP descriptor, representing the Wildman–Crippen LogP, indicated that low values negatively impacted pKi, though neutrality was also observed. High SLogP values, however, were predominantly associated with neutral effects. For the ATS1se descriptor, which is the Moreau–Broto autocorrelation of lag 1 weighted by Sanderson electronegativity, high values exhibited a neutral effect on pKi, while low values were associated with a positive influence, enhancing the predicted affinity. The NaaCH descriptor, which denotes the number of carbon atoms bonded by two aromatic bonds (typically within aromatic rings), displayed a more complex behavior. Low values of NaaCH positively influenced pKi, whereas high values had a negative effect. Nonetheless, both low and high values also demonstrated neutral contributions to pKi under certain conditions. The GGI7 descriptor, a seven-ordered raw topological charge, showed an ambiguous influence on pKi. Both low and high values were linked to negative or neutral effects without a clear pattern emerging from the data. The BIC2 descriptor, a two-ordered bonding information content, revealed a clearer trend. Low values were predominantly associated with a reduction in pKi, while high values exhibited a slightly positive effect, indicating a potential role in enhancing ligand affinity at higher descriptor values. For the JGI4 descriptor, a four-ordered mean topological charge, low values had a negative effect on pKi, while high values positively influenced the predicted affinity. Lastly, the SsssCH descriptor, representing the sum of carbon atoms bonded by three single bonds, exhibited a mixed pattern. Low values negatively influenced pKi, while high values showed a positive effect [30,34].

The results of the SHAP analysis summarized, given the ranges of the most important descriptors for the training set, formed the foundation for establishing the applicability domain. If at least seven ranges of the 10 descriptors are met, the molecule under test falls within the applicability domain, and then the prediction is defined as reliable.

#### 2.2.5. Case Study

To illustrate the mechanism of action of an antidepressant and validate model predictions for the activity of SSRIs and SNRIs, we chose Duloxetine as an example. Duloxetine is a registered drug belonging to the serotonin and norepinephrine reuptake inhibitor class. To obtain predictions, users need the SMILES representation of the molecule of interest. For Duloxetine, the SMILES string is shown below:

“CNCC[C@@H](C1=CC=CS1)OC2=CC=CC3=CC=CC=C32”.

**Figure 13:**
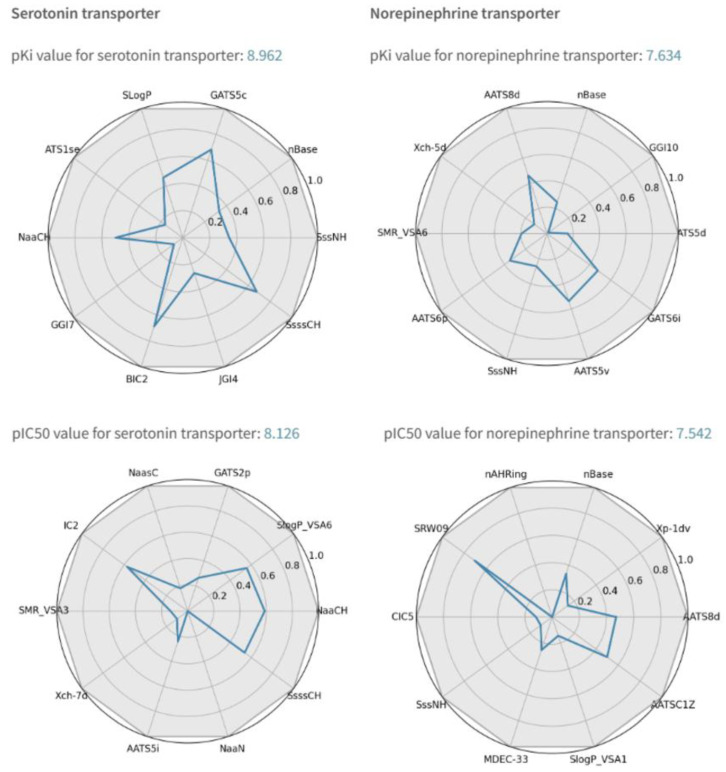
SerotoninAI predictions with applicability domain for Duloxetin.

The user simply needs to input the SMILES string into the designated cell in the SerotoninAI application. Within seconds, the results are displayed, as illustrated in Figure 13 below. The QSAR models predicted the following affinity values: pKi for SERT = 8.777 and pKi for NET = 7.757, which are very close to the experimental values reported in the literature (pKi for SERT = 9.1 and pKi for NET = 8.27) [35]. For the predicted pIC50 values, the application provided pIC50 for SERT = 8.126 and pIC50 for NET = 7.542. These predictions are consistent with the experimental values reported by Van Orden et al., where pIC50 for SERT = 8.2 and pIC50 for NET = 9.5 [36].

Additionally, the application provides information about the domain of applicability (see below). Duloxetine meets all predefined criteria, as its top 10 most important descriptors fall within the ranges defined by the training dataset. Finally, the application generates a comment regarding the potential use of the molecule within the appropriate mechanism, which is based on Table 7. For this case, the output states: “The molecule is a potential SSRI and SNRI drug”, which aligns with the known pharmacological properties of Duloxetine. Figure 13 shows the predictions and applicability domains for Duloxetin, which is a registered SNRI drug. The view shown is the result of the SerotoninAI application.

## 3. Discussion

In the presented work, we developed the general QSAR models to enable the search for new antidepressants that act like SSRIs or SNRIs. From the literature review presented in the introduction of the manuscript, to date, there are no models that consider the serotonin and norepinephrine transporter for both pKi and pIC50 values. The largest databases are a set of 213 ligands for the serotonin transporter [18] and 76 ligands for the norepinephrine transporter [7]. None of the models presented in the research articles is a simple user-accessible model.

Moreover, another aspect that underscores the relevance of our research is the lack of structural limitations of the obtained databases. They do not represent a set of molecules that are derivatives of one selected chemical structure, which stands in contrast to already published studies [7,8,9,10].

We also would like to mention the importance of developing two separate models for a given biological target for pKi and pIC50 values. An article from 2013 [37], in which the authors conduct an analysis of the ChEMBL database, suggests that the databases for pIC50 and pKi values should be merged to increase the size of the collection without losing data quality. The paper’s authors point out that there is a significant difference between the same molecules and their pIC50 values. A successive comparison of pIC50 and pKi values, after appropriate mathematical transformations, leads to values with similar error to pIC50 values only. In our opinion, consolidating the dataset in a single computational experiment would lead to unnecessary error. For this reason, we decided to limit the database to ligands whose differences in activity values do not exceed 100 nM and not to combine the databases of pIC50 and pKi values. In our research, we wanted to achieve the highest possible degree of consistency in the data, where experiments measuring inhibition and affinity values were carried out exclusively using human cell lines.

The models presented in this study have been implemented in a new module of the SerotoninAI application. The antidepressant activity module will allow the user to obtain a prediction and receive feedback on whether the molecule may show the potential of a future SSRI or SNRI drug in an accessible and quick way. In addition, the applicability domain is presented for each model.

## 4. Materials and Methods

### 4.1. Databases

The object of this manuscript is to develop QSAR models for the serotonin and norepinephrine transporters. The first step was to construct selected databases separately for these transporters and for two values of the inhibition constant (Ki) and the half-maximal inhibitory concentration (IC50). We used the ChEMBL [26], BindingDB [25], PubChem [27] and ZINC [28] platforms to curate as many compounds as possible. It was important to obtain ligands of these transporters with measured activity values based on human cell lines. Based on activity value conversions, the dependent variables in the study were pKi and pIC50.

### 4.2. Molecular Representation

The method of representing transporter ligands for machine learning methods to develop QSAR models was 2D Mordred descriptors [38]. Based on the experience gained with QSAR models for various serotonergic system databases, the chosen representation is the best form to train the model while accurately describing each descriptor [30]. The cleaning phase of the Mordred descriptors representing the ligands of biological targets consisted of removing columns with fixed values, and for columns that contained errors in the calculation of descriptors, the value was filled in with the column’s mean.

### 4.3. Machine Learning

Machine learning methods, particularly supervised machine learning, focus on training a model on a dataset so that it is both sufficiently fit to the training set and general enough to be applied to previously unseen data. In this way, they want to achieve a variance–bias trade-off. The experience gained [39] shows that the simplest methods of model development are insufficient to handle the complex nature of the ligand–protein relationship. We have developed QSAR models using the automated machine learning tool Mljar [40]. This system enables the development of complex ensemble models. The models were trained on 80% of the dataset according to 10-fold cross-validation (10-CV) and tested externally on the remaining set of ligands. Cross-validation is a method of evaluating and verifying model performance that is the gold standard for developing predictive models. The method involves dividing the data into several equal parts to repeatedly train and test the model on different subsets of the data.

### 4.4. Evaluation Metrics

We used the root mean square error (RMSE) and coefficient of determination (R^2^) to evaluate the obtained regression models. The calculations of these metrics are shown below in Equations (1) and (2). The best model was selected based on the lowest RMSE value.

(1) $$RMSE=\sqrt{\sum_{i=1}^{n}\frac{{\left({pred}_{i}-{obs}_{i}\right)}^{2}}{N}}$$

where RMSE is root mean square error, and N is the number of observations.

(2) $$R^{2}=1-\frac{{SS}_{res}}{{SS}_{tot}}=1-\frac{\sum_{i=1}^{n}{\left({pred}_{i}-obs\right)}^{2}}{\sum_{i=1}^{n}{\left({obs}_{i}-obs\right)}^{2}}$$

where R^2^ = the coefficient of determination, SS_res_ = the sum of squares of the residual errors, SS_tot_ = the total sum of the errors, obs_i_ and pred_i_ = observed and predicted values, and obs = arithmetical mean of observed values.

### 4.5. Model Interpretation

According to principle five of the Organization for Economic Cooperation and Development’s Guidelines for regulatory purposes on valid QSAR models, if possible, QSAR models should have a mechanistic interpretation [41]. A keyword that appears in relation to advanced machine learning models is Explainable Artificial Intelligence [42]. When using automated machine learning methods to develop QSAR models that have a high degree of advancement, the aspect of explainability of the entire model becomes difficult. However, a method that partially allows the interpretation of the results obtained and the influence of individual features on the prediction obtained is SHAP (Shapley additive explanations) analysis. The method is named after game theory inventor and Nobel Memorial Prize winner Lloyd Shapley. His considerations tested the influence of an individual participant in a team game on the final outcome [43,44].

For the top four QSAR models, the analysis was conducted based on a randomly selected 20% of the training set, using an adapted tool of SHAP analysis [45]. The obtained results formed the basis for establishing an applicability domain (AD), which informs the reliability of the prediction. When the molecule under test is at least within seven ranges of the training set values for the 10 most important descriptors for a given model, then such a prediction is reliable.

### 4.6. Software

Antidepressant activity is another module of the SerotoninAI application (https://serotoninai.streamlit.app/, accessed on 17 January 2025) [46]. The application was developed through the Streamlit platform and allows the user to easily obtain predictions of affinity and inhibitory concentration values for the serotonin and norepinephrine transporter. An additional aspect is the accompanying commentary for the user on whether the molecule could be a future drug in the SSRI or SNRI mechanism of action. Table 7 shows the ranges of pKi and pIC50 values and the concluding commentary. We would like to emphasize that the commentary is conditional, due to the need to limit the numerical values. The SerotoninAI source code and QSAR models are available on the GitHub platform (https://github.com/nczub/SerotoninAI_streamlit, accessed on 17 January 2025).

## 5. Conclusions

This study presents QSAR models for the serotonin and norepinephrine transporters, specifically predicting affinity (pKi) and inhibitory concentration (pIC50) values for experimentally measured unique sets of ligands. The developed models demonstrate low prediction errors and high coefficients of determination, indicating their robustness and reliability. These characteristics make the models valuable tools for application in the drug discovery process, particularly for identifying novel compounds with mechanisms of action as selective serotonin reuptake inhibitors (SSRIs) or serotonin–norepinephrine reuptake inhibitors (SNRIs).

## Figures and Tables

**Table 1:** Summary of databases for serotonin (SERT) and norepinephrine (NET) transporters.

Target	Property	ChEMBL	BindingDB	ZINC	PubChem (Without ChEMBL)	Molecules in Curated Database
NET	pIC50	1631	2136 (1486-ZINC; 1869-ChEMBL)	-	165	1879
NET	pKi	1598	2077 (1469-ZINC; 1390-ChEMBL)	3553	302	4304
SERT	pIC50	2827	3420 (2307-ZINC; 2782-ChEMBL)	-	533	2952
SERT	pKi	3059	3068 (2071-ZINC; 2263-ChEMBL)	7312	615	8872

**Table 2:** Summary of results for QSAR models of serotonin and norepinephrine transporters.

Target	Property	RMSE 10-CV	R^2^ 10-CV	RMSE Test	R^2^ Test	Ensemble Model Structure
NET	pIC50	0.621	0.708	0.678	0.640	2 × Xgboost, 5 × LightGBM, Neural Network, 3 × CatBoost
NET	pKi	0.522	0.764	0.590	0.709	3 × Xgboost, 2 × LightGBM, 3 × Neural Network, CatBoost
SERT	pIC50	0.597	0.715	0.645	0.678	Xgboost, 4 × LightGBM, 4 × Neural Network
SERT	pKi	0.474	0.863	0.540	0.828	3 × Xgboost, 7 × LightGBM, 6 × Neural Network, 2 × CatBoost

**Table 3:** Shapley values for ten most important descriptors for the NET-pIC50 QSAR model.

Descriptor	Description [30]	Shapley Value
AATS8d	Averaged Moreau–Broto autocorrelation of lag 8 weighted by sigma electrons	0.08196
Xp-1dv	1-ordered Chi path weighted by valence electrons	0.06080
nBase	Basic group count	0.05944
nAHRing	Aromatic hetero ring count	0.05231
SRW09	Walk count (leg-9, only self-returning walk)	0.04940
CIC5	5-ordered complementary information content	0.04560
SssNH	Sum of nitrogen atoms bonded by two single bonds	0.04211
MDEC-33	Molecular distance edge between tertiary C and tertiary C	0.04023
SlogP_VSA1	MOE logP VSA Descriptor 1 (-inf < x < −0.40)	0.03818
AATSC1Z	Averaged and centered Moreau–Broto autocorrelation of lag 1 weighted by atomic number	0.03776

**Table 4:** Shapley values for ten most important descriptors for NET-pKi QSAR model.

Descriptor	Description [30]	Shapley Value
ATS5d	Moreau–Broto autocorrelation of lag 5 weighted by sigma electrons	0.05284
GGI10	10-ordered raw topological charge	0.04617
nBase	Basic group count	0.04613
AATS8d	Averaged Moreau–Broto autocorrelation of lag 8 weighted by sigma electrons	0.04608
Xch-5d	5-ordered Chi chain weighted by sigma electrons	0.04140
SMR_VSA6	MOE-type descriptors using Wildman–Crippen MR and surface area contribution	0.04028
AATS6p	Averaged Moreau–Broto autocorrelation of lag 6 weighted by polarizability	0.03630
SssNH	Atom type e-state descriptor—sum of nitrogen atoms bonded by two single bonds	0.03408
AATS5v	Averaged Moreau–Broto autocorrelation of lag 5 weighted by vdw volume	0.02857
GATS6i	Geary coefficient of lag 6 weighted by ionization potential	0.02807

**Table 5:** Shapley values for ten most important descriptors for SERT-pIC50 QSAR model.

Descriptor	Description [30]	Shapley Value
NaaCH	Number of aromatic carbon atoms	0.06192
SlogP_VSA6	MOE logP VSA Descriptor 6 (0.15 ≤ x < 0.20)	0.04926
GATS2p	Geary coefficient of lag 2 weighted by polarizability	0.04684
NaasC	Number of aromatic carbon atoms with single bond	0.04545
IC2	2-ordered neighborhood information content	0.04496
SMR_VSA3	MOE Molar Refractivity VSA Descriptor 3 (1.82 ≤ x < 2.24)	0.04269
Xch-7d	7-ordered Chi chain weighted by sigma electrons	0.04099
AATS5i	Averaged Moreau–Broto autocorrelation of lag 5 weighted by ionization potential	0.03823
NaaN	Number of nitrogen aromatic atoms	0.03417
SsssCH	Sum of tertiary carbon atoms	0.03109

**Table 6:** Shapley values for ten most important descriptors for SERT-pKi QSAR model.

Descriptor	Description [30]	Shapley Value
SssNH	Atom type e-state descriptor—sum of nitrogen atoms with two single bonds	0.09159
nBase	Basic group count	0.08458
GATS5c	Geary coefficient of lag 5 weighted by Gasteiger charge	0.05913
SLogP	Wildman–Crippen LogP	0.05444
ATS1se	Autocorrelation of Topological Structure descriptor—Moreau–Broto autocorrelation of lag 1 weighted by Sanderson EN	0.05037
NaaCH	Atom type e-state descriptor—number of aaCH	0.04245
GGI7	7-ordered raw topological charge descriptor	0.04082
BIC2	2-ordered bonding information content	0.04035
JGI4	4-ordered mean topological charge	0.04015
SsssCH	Atom type e-state descriptor—sum of tertiary carbon atoms	0.03947

**Table 7:** Predictions values of pKi and pIC50 for serotonin and norepinephrine transporters and corresponding comment from SerotoninAI application.

NET pKi	NET pIC50	SERT pKi	SERT pIC50	Comment
≥7	≥7	≥7	≥7	Molecule is a potential SSRI and SNRI drug
		≥7	≥7	Molecule is a potential SSRI drug
≥6 & <7	≥6 & <7	≥6 & <7	≥6 & <7	Molecule might have SSRI and SNRI mechanism of action
		≥6 & <7	≥6 & <7	Molecule might have SSRI mechanism of action
<6	<6	<6	<6	The molecule under study does not exhibit antidepressant activity by the mechanism of SSRIs and SNRIs

## Data Availability

The data presented in this study are available on request from the corresponding author.

## Supplementary Materials

The following supporting information can be downloaded at https://www.mdpi.com/article/10.3390/molecules30030637/s1. In the Supplementary Materials, the authors share four databases of pKi and pIC50 values for serotonin and norepinephrine transporters S1–S4.
